# Supplementary figures and images for: A malaria parasite phospholipase facilitates efficient asexual blood stage egress
Source: PLoS Pathog. 2023 Jun 23;19(6):e1011449. doi: 10.1371/journal.ppat.1011449 (PMC10325081; doi:10.1371/journal.ppat.1011449)

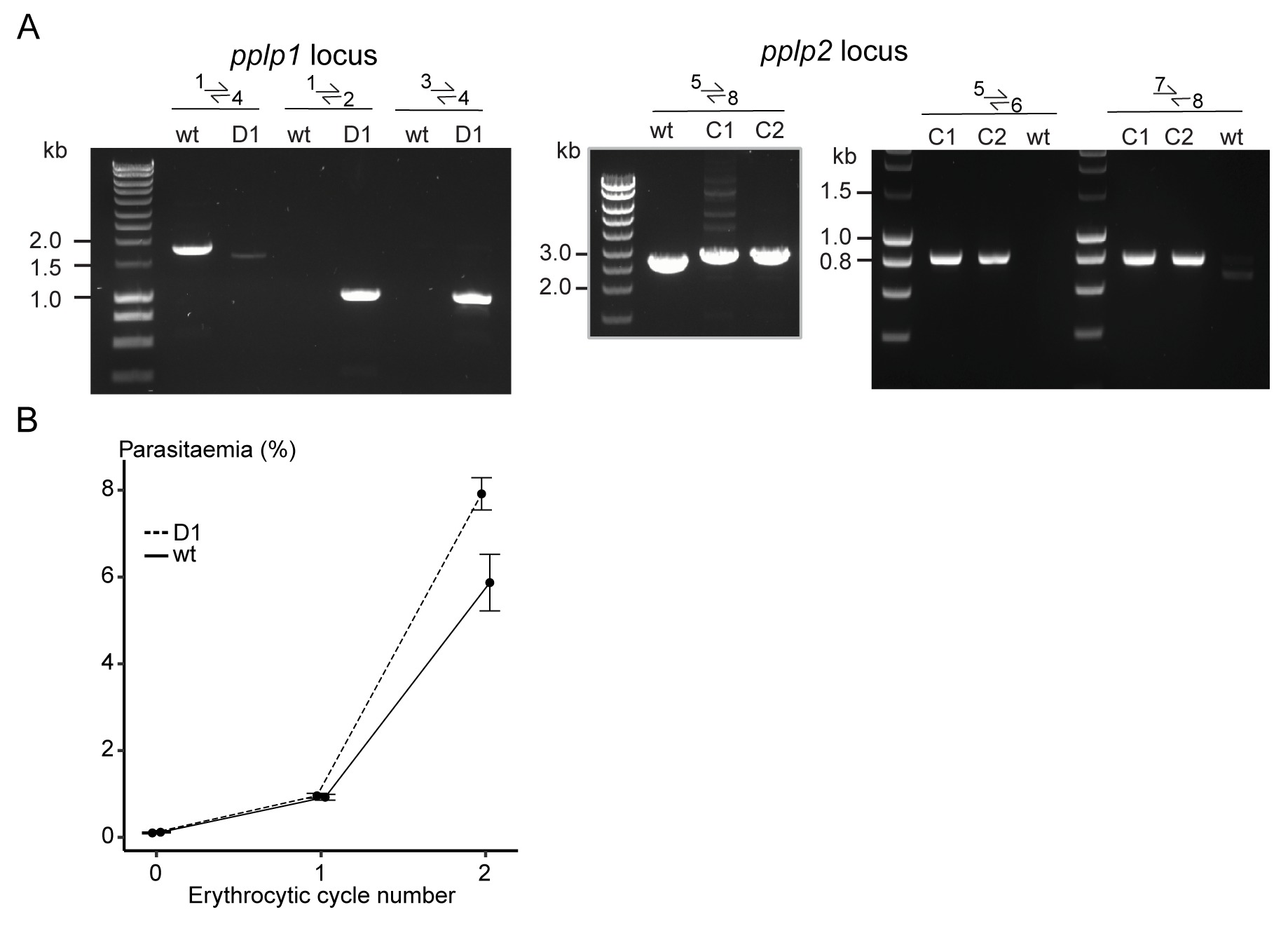

Supplement: S1 Fig — A) Diagnostic PCR showing correct integration of the modification plasmids into the PPLP1 and PPLP2 loci in PPLP1:loxNint/PPLP2:loxPint parasites. Primers used are denoted in Fig 1A. B) Replication of PLP1:loxPint clonal line prior to second modification. The modified parasites show a normal replication rate across two cycles (error bars, ± SD, triplicate RAP treatments with different blood sources). (TIF) [file ppat.1011449.s001.tif]

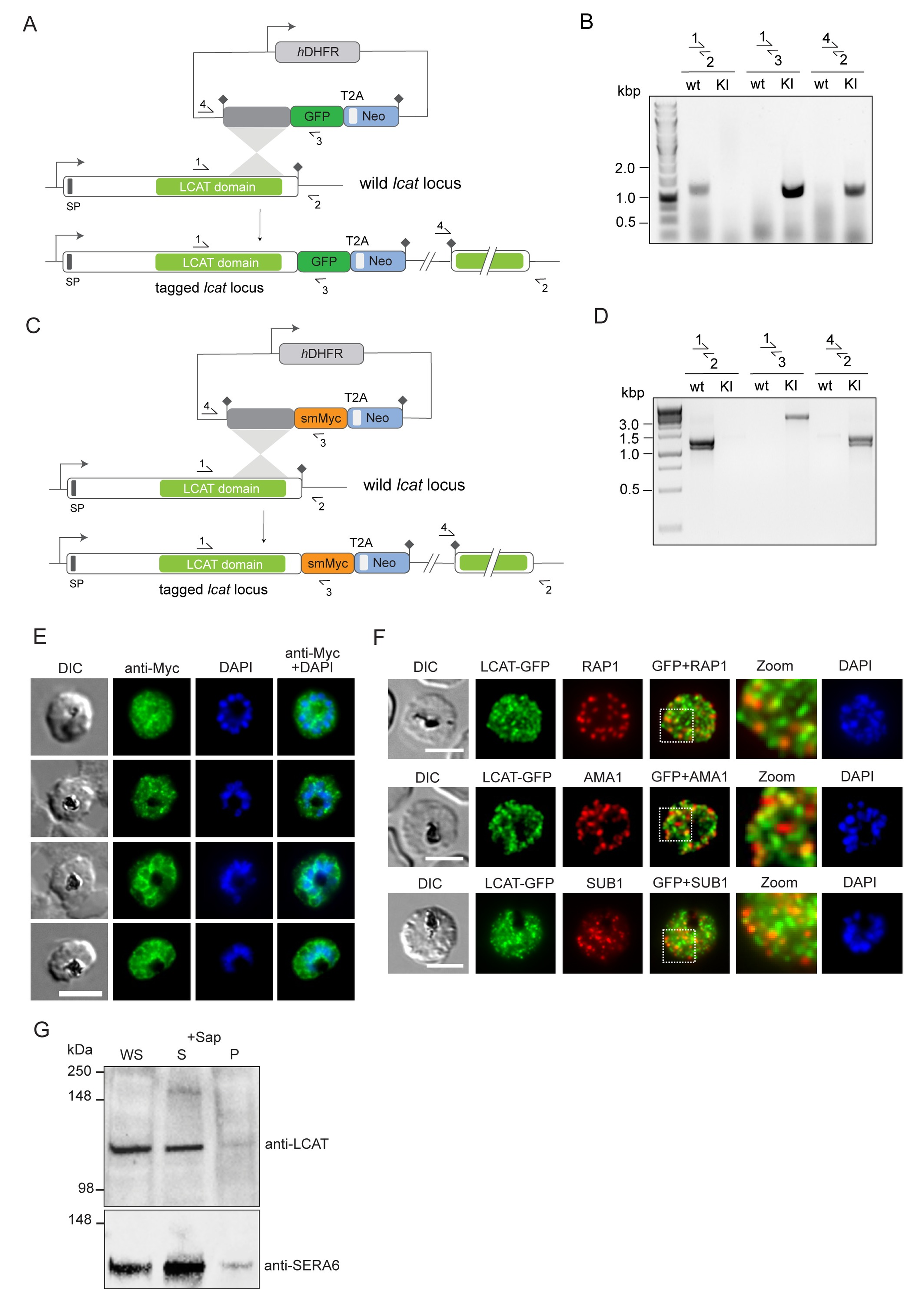

Supplement: S2 Fig — A) Strategy for SLI-based endogenous tagging of the lcat gene with GFP. Primers used for integration PCR are indicated with half arrows. T2A, skip peptide; Neo-R, neomycin-resistance gene; hDHFR, human dihydrofolate reductase; lollipop, stop codons; arrows, promoters. B) Diagnostic PCR showing correct integration of the modification plasmid into the LCAT locus in the LCAT:GFP parasites. KI, knock in cell line; WT, wild type parental line. C) Strategy for SLI-based endogenous tagging of lcat gene with smMyc. Primers used for integration PCR are indicated with half arrows. D) Diagnostic PCR showing correct integration of the modification plasmid into the LCAT locus in the LCAT:smMyc line. E) IFA of LCAT:smMyc mature schizonts using anti-myc (green) antibodies showing similar localisation of LCAT as observed in LCAT:GFP line (Fig 3). DAPI-stained nuclei are shown in blue. DIC, differential interference contrast. Scale bar, 5 μm. F) Colocalisation analysis of LCAT:GFP (green) with markers for rhoptries (RAP1), micronemes (AMA1) and exonemes (SUB1) in C2-arrested schizonts. DAPI-stained nuclei are shown in blue. DIC, differential interference contrast. Scale bar, 5 μm. G) Western blot using anti-LCAT polyclonal antibodies shows distribution of LCAT upon saponin-mediated fractionation (using treatment with 0.05% saponin at 37°C for 5 min) of schizonts. LCAT was predominantly detected in the supernatant fraction (+SAP “S”) compared to the pellet fraction (+SAP “P”), similar to the soluble PV marker SERA6. The appropriate band size of LCAT is shown from whole schizonts (WS) for comparison (same lane also shown as -RAP in Fig 4C). (TIF) [file ppat.1011449.s002.tif]

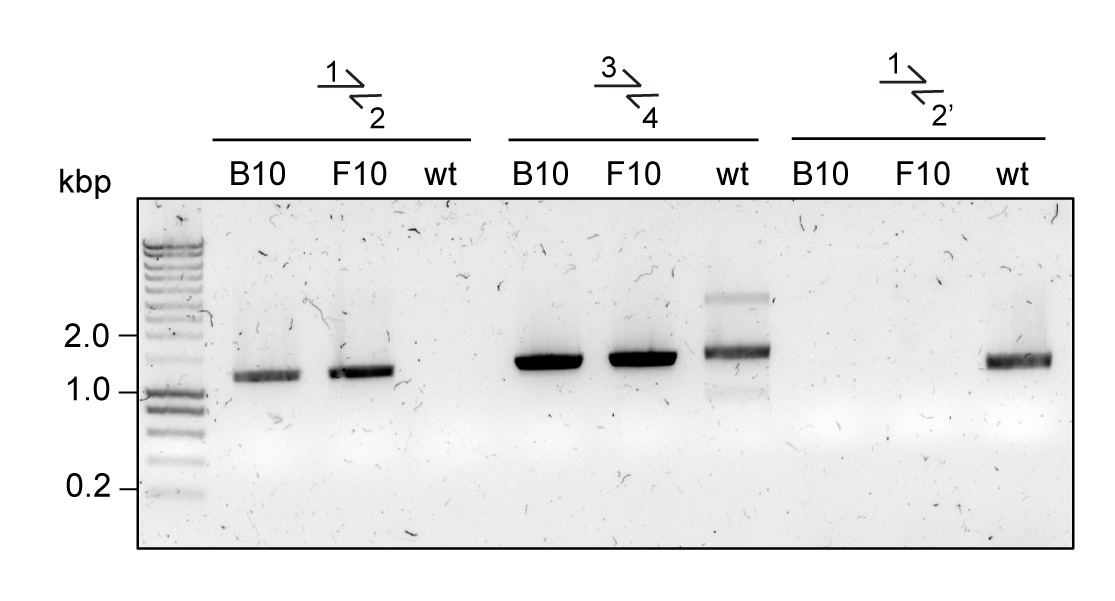

Supplement: S3 Fig — A) Diagnostic PCR showing correct integration of the modification plasmid into the LCAT locus in LCAT:2loxPint line. Primers used are indicated in Fig 4A. (TIF) [file ppat.1011449.s003.tif]

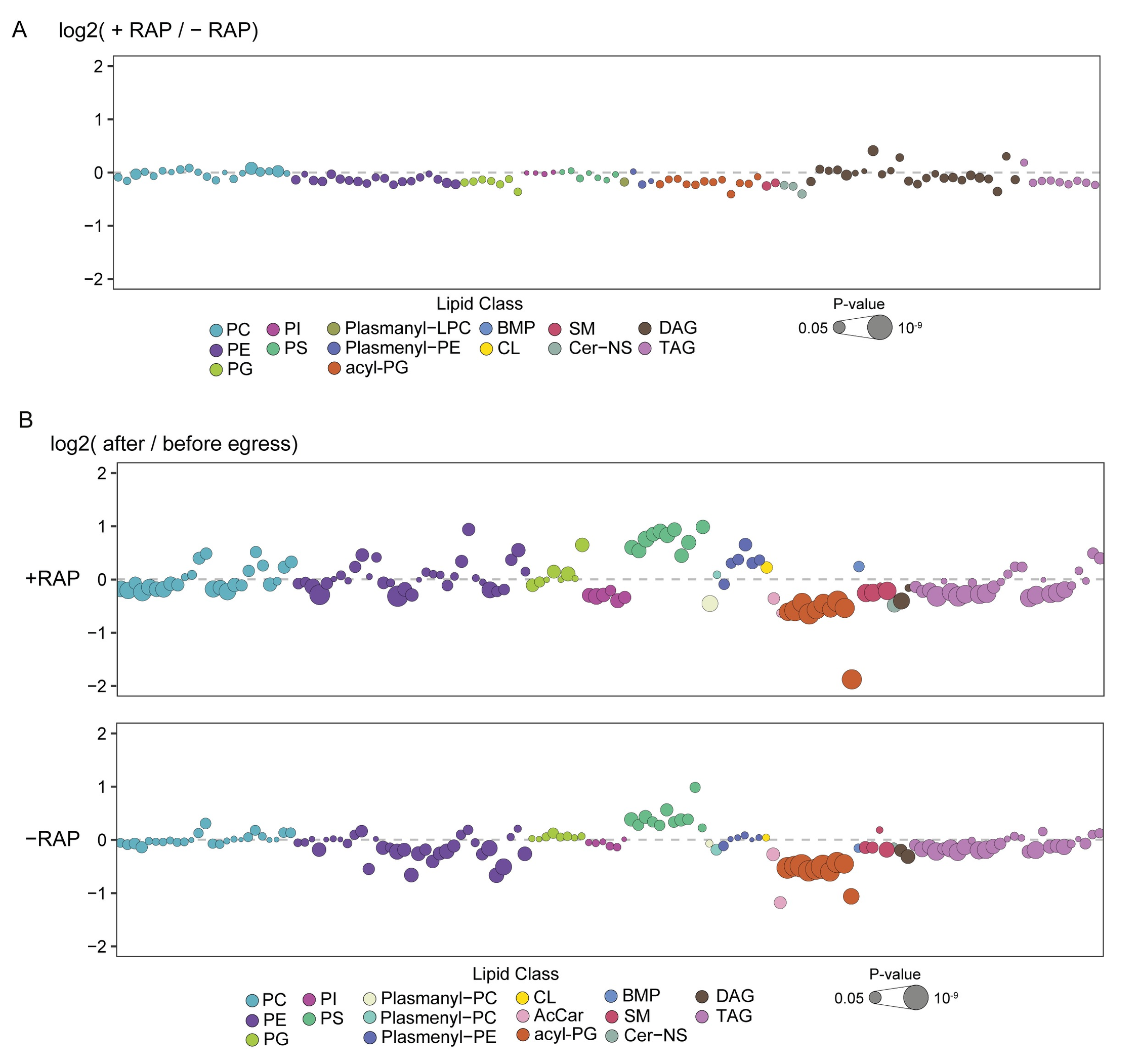

Supplement: S4 Fig — A) Lipidomic analysis of LCAT:2loxPint egress-stalled schizonts following mock-or RAP-treatment at ring stages. The bubble plot shows the fold change (y-axis) in levels of various lipid species (each lipid class denoted with a different colour) in LCAT-null schizonts compared to controls (3 independent biological replicates). No significant change in phospholipid levels were detected between the samples. B) Bubble plot showing the fold change in levels of various lipid species before and after egress of RAP-treated (+RAP) and mock-treated (-RAP) LCAT:2loxPint parasites (6 independent biological replicates). (TIF) [file ppat.1011449.s004.tif]

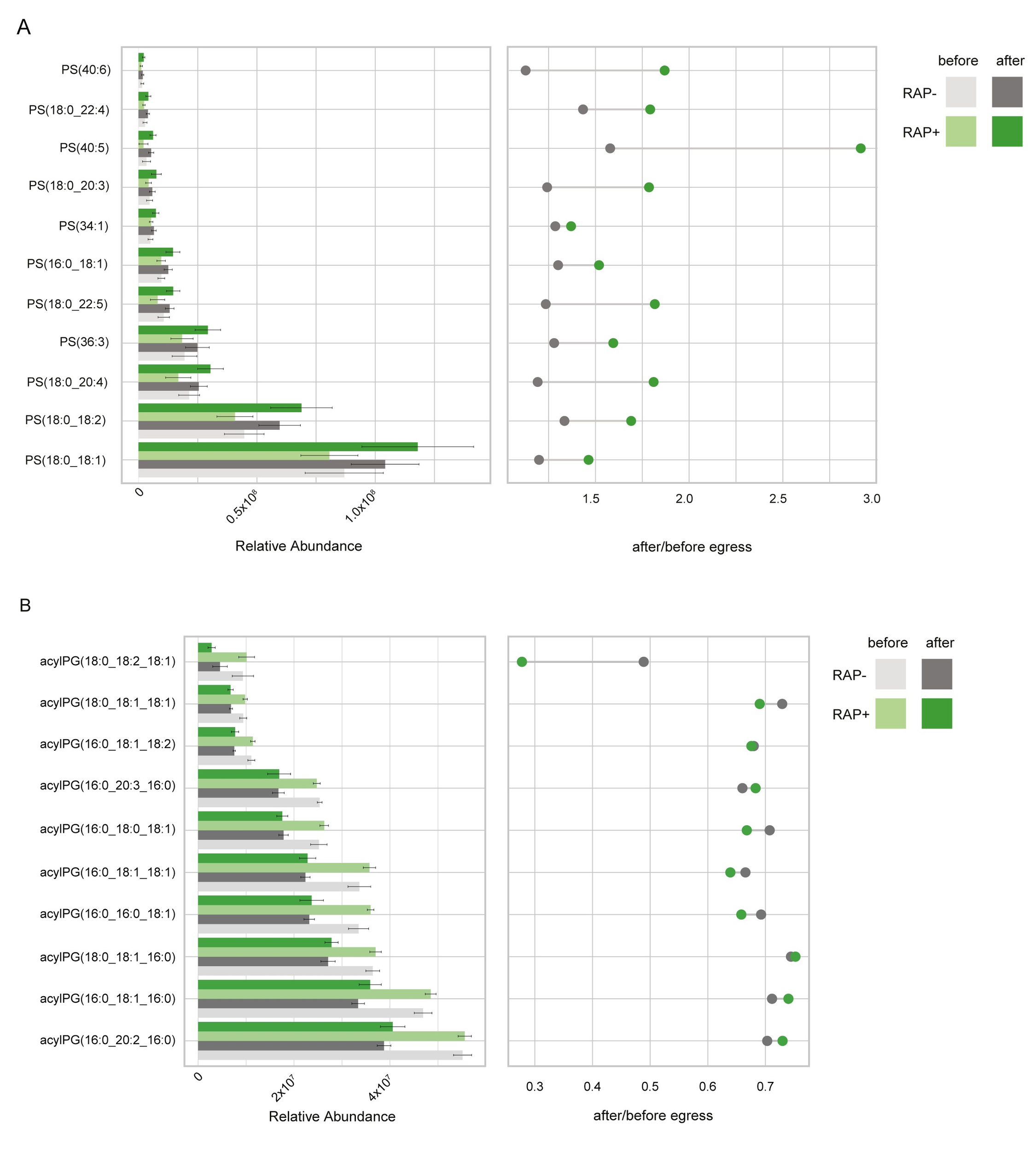

Supplement: S5 Fig — Relative peak intensities (depicted as barplots) and log2 fold change (depicted as dot plots) of the significantly altered A) phosphatidylserine and B) acylphosphatidylglycerol species upon egress of mock- or RAP-treated LCAT:2loxPint schizonts. (TIF) [file ppat.1011449.s005.tif]
