# Supplementary material for: A malaria parasite phospholipase facilitates efficient asexual blood stage egress
Source: PLoS Pathog. 2023 Jun 23;19(6):e1011449. doi: 10.1371/journal.ppat.1011449 (PMC10325081; doi:10.1371/journal.ppat.1011449)
Supplement: Attachment — Submitted filename: ResponseToReviewers_final.docx [file ppat.1011449.s013.docx]

**Part I – Summary**

Please use this section to discuss strengths/weaknesses of study, novelty/significance, general execution and scholarship.

Reviewer #1: The manuscript by Ramaprasad and colleagues investigates the role two perforin-like proteins in Plasmodium falciparum egress. After demonstrating that PLP1 and PLP2 are not required, the group more deeply investigates the role of a lecithin:cholesterol acyltransferase protein (LCAT). LCAT knockout led to reduced parasite growth with resulting changes in lipid profile of parasites.

The use of two different Cre recombinase sites, loxP and loxN, is a nice way to delete the two PLP genes at the same time without interference. As noted, this was not necessary as they inadvertently frameshifted PLP1 during the first transfection. The dual deletion parasite strain demonstrates that neither PLP1 or 2 are needed for asexual replication.

The proteomic approach to identify microneme/exoneme proteins uses SUB1-null parasites in the presence and absence of C2. This was the starting point to investigate LCAT in asexual parasites. The phenotyping of the LCAT mutant is interesting and suggests a difference in the lipid composition. However, no follow up studies are performed to verify this finding. Therefore, the reader is left without knowing what the impact of the increased PS and PE species in LCAT-null parasites means for parasite egress.

The findings of the study are different from the initial studies of LCAT in P. berghei where Bhanot, et al. reported no major growth defects in asexual parasites. However, the finding of delayed egress and impaired PVM rupture is the same phenotype as described in Pb liver stage parasites by Burda in 2015. In this second paper, a major defect in asexual growth was also not described.

Reviewer #2: Egress from infected red blood cells is a critical step in the lifecycle of malaria parasites. Although a variety of parasite egress factors have been identified for this event, the known factors cannot account for all the observed events that accompany egress. Previous reports implied that perforin-like proteins 1 and 2 might be expressed in late stage schizonts and contribute to egress in a manner shown for other stages of the parasite and related parasites. The current study advances knowledge of this topic by: (1) definitively showing that PfPLP1 and PfPLP2 are not necessary for normal egress; (2) developing an innovative proteomics screen that identified secretory proteins that are released into the supernatant during egress, including a phospholipase, LCAT, that has been partially characterized previously; (3) showing that conditional KO of LCAT markedly reduces efficient egress, thereby blunting proliferation; and (4) identifying PS and PE as phospholipids that are depleted in an LCAT-dependent manner during egress. By shifting the focus on pore-forming proteins to a phospholipase, this study provides a new perspective on how malaria parasites exit from cells to advance the infection. Addressing the following comments should further strengthen the study.

Reviewer #3: In this paper, the authors investigated molecular events involved in asexual blood stage egress of the deadly parasite Plasmodium falciparum.
This topic is really important in the apicomplexa field but also for the parasite community in general.
As a general comment, the paper is well written, experiments are well presented and done rigorously with appropriate controls and humility in their interpretation.
Even if the authors did not solve completely their problematics they pointed out several issues that will help to better understand the topic but also, with no doubts, will serve for future studies.
4 main points are raised:
-Two performing-like proteins are not involved in egress
-Identification of proteins discharged in the parasitophorous vacuole during egress
-Potent role of a phospholipase in the egress
-Role of acyl-phosphatidyl glycerol during egress.
Strenghts: robust and elegant experiments adding more pieces in the understanding of egress
Weakness: The paper is not really focused.... the way the authors explain why their work on a phospholipase is not intuitive..and after in some extent "negative" results.
The title do not reflect properly the content of the paper. I would suggest to modify the title. In my point of view, the focus on the phospholipase is not justified as it is only part of the demo.

We agree with the reviewer that the paper is not completely focussed on the phospholipase LCAT, but we are sure that the reviewer appreciates that a succinct title that additionally summarises our work with the perforin-like proteins would be difficult to devise. As the reviewer rightly points out, we landed on LCAT after pursuing different lines of enquiry, some of which resulted in “negative” data in that they rule out a role for PPLP1 and PPLP2 in egress. We felt it was important to report these results in order to correct previous potentially misleading models in the field regarding these perforin-like proteins and to inform future studies on what has been tried. We explain our rationale at the outset of each section of the manuscript so that the reader can follow the progress of our thinking throughout the study. As we eventually were able to identify one molecule with a clear role in egress, LCAT, we believe the title of the paper should reflect that main finding in lieu of a more generic title.

**Part II – Major Issues: Key Experiments Required for Acceptance**
Please use this section to detail the key new experiments or modifications of existing experiments that should be absolutely required to validate study conclusions.

Generally, there should be no more than 3 such required experiments or major modifications for a "Major Revision" recommendation. If more than 3 experiments are necessary to validate the study conclusions, then you are encouraged to recommend "Reject".

Reviewer #1: MAJOR:
1. The localization data shown (fig 3a, 3b, and sup 2) should be improved some. The hypothesis, which is very likely to be correct, is that LCAT is transported through the secretory system and resides in the PV. The immunofluorescence does not show this clearly. The LCAT and SERA5 staining are diffuse and overlap with MSP1. As shown, the staining could also be interpreted as cytoplasmic. The other data strongly support the hypothesis that LCAT is in the PV. The immunofluorescence should be improved to strengthen this hypothesis even more.

We thank the reviewer for this suggestion. Unfortunately, despite several attempts, we were unable to get a good signal from IFA with the anti-LCAT polyclonal antibody, presumably due to low LCAT abundance. As a result, we had to capture the signal with increased laser intensity or exposure. While this was helpful to show LCAT ablation in Fig 4D, it was not suitable to show clear co-localization with SERA5, as the reviewer rightly points out. We have now removed this low quality data from Fig 3 to avoid confusion. Instead, we have now added a western blot (Supplementary Figure S2G) using the same antibody that shows most of the LCAT being released into the supernatant upon saponin lysis of schizonts, consistent with its PV localisation. We hope that the independent lines of evidence from three different parasite lines – LCAT-GFP (live GFP signal in Figure 3), LCAT-smMyc (anti-myc IFA in Supplementary Figure 2E) and LCAT-2loxPint (anti-LCAT western blot in Supplementary Figure S2G) strongly supports our hypothesis. New sentences have now been added to page 9 and 10 of the revised manuscript to reflect these changes.

2. The egress phenotype is described as abnormal. The meaning of abnormal needs to be stated so that the reader can understand the quantification in 5D. Similarly, for 5B, what percentage of the egress events had joined merozoites? This is presumably what is being quantified in 5D, but this is not stated.

We describe the abnormal phenotype thus on page 11: “In contrast, LCAT-null schizonts showed clear delays between these sequential events, with reduced merozoite release upon RBCM rupture, the remaining merozoites often appearing clumped together (Figure 5B-D, Supplementary Movie S2).”

We agree with the reviewer, however, that the definition of “abnormal” which we use at several places is not obvious. In our revised manuscript we have therefore now changed the above sentence to be more explicit:

“In contrast, LCAT-null schizonts showed **an abnormal egress phenotype** with clear delays between these sequential events, with reduced merozoite release upon RBCM rupture, the remaining merozoites often appearing clumped together (Figure 5B-D, Supplementary Movie S2).”

The reviewer is also right that egress events with clumped merozoites as shown in Figure 5B is what is being quantified in Figure 5D. We have added the following sentence to 5D legend to be clear: “We defined an egress event as abnormal when only a few merozoites managed to disperse upon RBCM rupture while the remaining merozoites remained clumped together.” We hope that these revisions clarify this issue.

3. Would it be interesting to compare the after egress -RAP to the after egress +RAP to look for differences that are specific to LCAT? The current analysis looks at after/before egress for each condition. Also, for the lipid analysis, it would be helpful to include some additional description in the text of what the enzymatic function of LCAT is, what types of lipids it is expected to modify, etc. For the non-expert, the meaning and importance of the lipid analysis and the changes is tough to appreciate. If the changes are important, this is not fully conveyed in the text.

We did indeed compare those groups as we, like the reviewer, thought it would be interesting. However, this analysis did not detect any significant changes (find below a volcano plot showing the comparison). This perhaps is not surprising as it reflects the lack of any difference in lipid composition between -RAP and +RAP schizonts that we show in Figure S4A.

In our manuscript, we do discuss the known enzymatic function of LCAT and the possible significance of our lipidomics results on pages 18-19. Briefly, while LCAT is known to hydrolyse PC to lysoPC, our lipidomics results do not show any significant changes in PC or lysoPC levels. We believe that any changes resulting from LCAT depletion are minimal and under the range of detection by our lipidomics approach. Canonical LCAT enzyme is not known to act on PS, the phospholipid which does change during LCAT-null schizont egress. Therefore, we think the change in PS levels is most likely a result of the abnormal egress phenotype rather than a direct result of loss of LCAT activity. We hope these views are made clear in our revised manuscript.

Reviewer #2: Main comments
1. The authors conclude that LCAT localizes to secretory vesicles. However, the evidence for this is limited to one fluorescent micrograph showing the distribution of LCAT-GFP possibly being within the parasite and a second micrograph indicating possible relocation of LCAT-GFP to the PV. The authors presumably have good antibodies to microneme, rhoptry, and exoneme markers that could better define where LCAT resides within the parasite. Doing so would help broaden understanding the contributions of various secretory organelles to egress.

We thank the reviewer for these excellent suggestions. In addition to the micrograph of LCAT-GFP in Figure 3, we would like to draw attention to the additional evidence in Supplementary Figure S2E where IFAs with a separate line LCAT- smMyc shows again LCAT localisation to focal structures and PV.

To further address this issue, in our revised manuscript we have now included co-localization data with various organellar markers as Supplementary Figure S2F. These experiments showed that LCAT-GFP did not co-localize with RAP1 (rhoptry), AMA1 (micronemal) or SUB1 (exoneme) markers. New sentences have now been added to pages 9 and 10 to reflect these changes.

2. Related to point #1, data from screens is typically validated by performing a similar experiment that more selectively looks at the target of interest. In this manner it would be ideal to redo the C1/saponin experiment and test s/n’s for LCAT (untagged) and LCAT-GFP. Comparing pellets to S/N’s would also allow assessment of potential proteolytic processing of LCAT as a possible means of regulating its activity.

We thank the reviewer for this suggestion. We have now added a western blot (Supplementary Figure S2G) that shows most of the LCAT being released into the supernatant upon saponin lysis of schizonts, consistent with its putative PV localisation. We have also performed several experiments to assess proteolytic processing of LCAT, but unfortunately the results were not consistent. As a result, we did not include this data in the paper. New sentences have now been added to pages 9 and 10 to reflect these changes.

Reviewer #3: (No Response)

**Part III – Minor Issues: Editorial and Data Presentation Modifications**
Please use this section for editorial suggestions as well as relatively minor modifications of existing data that would enhance clarity.

Reviewer #1: MINOR
1. There are formatting errors for the references. The Burda bioRxiv paper is present twice (2021a and 2021b).

This is now corrected.

2. The source of the SUB1-null parasites is not discussed. Is this an inducible SUB1 knockout?

We apologise for this omission. On page 8 of the revised manuscript we have now amended the text to: “To selectively identify these ‘trapped’ components, we allowed SUB1-null schizonts (created by inducing ablation of SUB1 expression in the inducible knockout line, SUB1HA3:loxP (Thomas *et al.*, 2018)) to mature in the presence of C2……”

3. The identity of the remaining significant red dot proteins should be indicated in figure 2c. Currently there are identifications provided for only a subset of the proteins.

We have now labelled all the red dots and the dot corresponding to LCAT.

4. Fig 4F seems to provide more information than is valuable for the reader. Although briefly explained in the legend, the meaning of different features of the graph remains unclear. This is strong data showing the replication defect. It should be presented in a more straightforward manner.

The graph (a raincloud plot) is designed to represent the data in an unbiased and transparent manner. We have now removed some confounding shades and gridlines and also reduced the size of the datapoints for better visibility.

5. Page 11 “solely ascribed” is a strong phrase. This should be worded less definitively.

Changed to “be primarily ascribed to”

6. Fig 5F, the image looks strange. The phalloidin image does not look homogenous in the field of view. This makes interpretation difficult. This should be repeated, potentially with better alignment or adjustment.

Unfortunately, this is an issue with the alignment of our microscope which we have so far been unable to correct. However, since we only count the poration events that we can visualise, we do not believe that this in any affects our quantitation of poration or the reliability of our data (since it is effectively equivalent to using an imaging set-up with a smaller field of view). We trust that the reviewer accepts this point.

7. In Supp movies, the use of Hoechst staining to differentiate the two conditions is clever. This should be stated somewhere besides the legend for the supplemental movies. I needed to watch the movies, consider what was going on, then re-read the legends again. This is minor, but it would be helpful to mention this in the text as well.

We really appreciate this valuable comment from a reader’s standpoint. Accordingly, we have now altered the text in the Results to:

Page 7: “To assess RBCM poration, we performed simultaneous time-lapse imaging of both RAP- and mock-treated (stained blue with DAPI) PPLP1: loxNint/PPLP2:loxPint parasites in the presence of fluorescent phalloidin (green), a cyclic peptide that gains access and binds to the RBC cytoskeleton upon RBCM poration. These experiments showed that the PPLP1/PPLP2-null schizonts underwent typical RBC poration just prior to egress (Supplementary Movie S1).”

Page 11: “We then visualised the behaviour of RAP- (stained blue with Hoechst) and mock-treated LCAT:2loxPint schizonts as they underwent egress using time-lapse video microscopy.”

and

“These experiments showed that whilst RBCM poration did take place in LCAT-null parasites (stained with Hoechst), we observed a small reduction in the proportion that had undergone poration, suggestive of a slightly slower rate of poration in the mutants”

Reviewer #2: Minor comments
Page 4, 2nd line from bottom: citation appears to be incorrect.

The citation is correct; it is a two-author review article on Apicomplexan pore-forming proteins. The individual research articles associated with each PPLP are cited in subsequent sentences.

Page 7, 3rd paragraph, 2nd line: “…no effect on parasite proliferation…” Consider being more precise here and use the term “egress” instead of “proliferation” if the studies assessed egress.

Wirth *et al.,* 2014 studied not just egress but also invasion and parasite development during blood stages (and found them to be normal). Yang *et al.,* 2017 on the other hand only assessed blood stage growth fitness and found it to be normal. Therefore, to convey all these findings briefly, we use “parasite proliferation” here. We hope this is acceptable.

Page 9, 1st paragraph, 7th line: should singular/plural misalignment. Should be candidate and was or candidate and were.

This text has now been changed to “but no candidates with established membranolytic activity were identified”

Fig 2b. Please label the dot corresponding to LCAT. Also, since MSP1 and MSP2 were identified as being significantly enriched, the authors should mention this and disclose that not all of the proteins identified were from apical secretory organelles.

In the revised manuscript have now labelled all the red dots and the dot corresponding to LCAT. We have also added the following sentence to the Results:

“Apart from a few surface (MSP1 and MSP2), rhoptry neck (RH2a) and PV (PTEX88) proteins, these included the exoneme protease plasmepsin X (PMX) (Nasamu et al., 2017; Pino et al., 2017) which was enriched 4-fold, as well as four membrane proteins that are discharged from micronemes during schizont rupture: EBA-140, EBA-181, GAMA and MTRAP (4-fold to 24-fold enrichment).”

Page 9, 2nd paragraph, 3rd line: “To confirm the localization…” The expression data doesn’t indicate localization. Should be “To investigate the localization…”

This is now altered to “To investigate..”

Page 19, 1st paragraph, last line: the importance of acyl-PG is not addressed in the study. Rather, the abundance of this acyl-PG decreases during egress. Additional work would be necessary to define its importance.

The reviewer makes a good point. We have changed this phrase to “…identified acyl-PG as an important phospholipid that undergoes substantial depletion during asexual blood stage egress.”

Reviewer #3: The authors should detail more the caption in the figures and order to facilitate the understanding, in particular in the movies.

The authors should give more details in the analysis of MS data. The link between the data in Supplementary Table S2 and the volcano plot is not obvious. Give more details.

These are good suggestions. All the captions now hold more detailed information regarding what is being visualized, which group has been stained, etc. Supplementary Table S2 of the revised manuscript now comprises two sheets: “Analysis” which shows details of t-test comparisons and “Rawdata” which shows the normalized intensity values.

**Other changes**

The Supplementary Videos have been updated to increase resolution and add time stamps.
